# The burden of excessive saturated fatty acid intake attributed to ultra-processed food consumption: a study conducted with nationally representative cross-sectional studies from eight countries

**DOI:** 10.1017/jns.2021.30

**Published:** 2021-06-04

**Authors:** Eurídice Martínez Steele, Carolina Batis, Gustavo Cediel, Maria Laura da Costa Louzada, Neha Khandpur, Priscila Machado, Jean-Claude Moubarac, Fernanda Rauber, Marcela Reyes Jedlicki, Renata Bertazzi Levy, Carlos A. Monteiro

**Affiliations:** 1 Department of Nutrition, School of Public Health, University of São Paulo, São Paulo, Brazil; 2 Center for Epidemiological Studies in Health and Nutrition, University of São Paulo, São Paulo, Brazil; 3 CONACYT – Center for Nutrition and Health Research, National Institute of Public Health, Mexico City, Mexico; 4 School of Nutrition and Dietetics, University of Antioquia, Medellín, Colombia; 5 Department of Nutrition, Harvard T.H. Chan School of Public Health, Boston, USA; 6 Institute for Physical Activity and Nutrition (IPAN), School of Exercise and Nutrition Sciences, Deakin University, Geelong 3220, Australia; 7 Département de Nutrition, Université de Montréal, Montréal, Canada; 8 Department of Preventive Medicine, School of Medicine, University of São Paulo, São Paulo, Brazil; 9 CIAPEC, Unidad de Nutrición Pública, INTA, Universidad de Chile, Santiago, Chile

**Keywords:** Diet, Population attributable fraction, Saturated fatty acids, Ultra-processed foods, CI, confidence interval, CVD, cardiovascular disease, ES, estimated size, NCD, non-communicable disease, PAF, population attributable fraction, PR, prevalence ratio, UPF, ultra-processed food

## Abstract

Cross-sectional nutritional survey data collected in eight countries were used to estimate saturated fatty acid intakes. Our objective was to estimate the proportion of excessive saturated fatty acid intakes (>10 % of total energy intake) that could be avoided if ultra-processed food consumption was reduced to levels observed in the first quintile of each country. Secondary analysis was performed of 24 h dietary recall or food diary/record data collected by the most recently available nationally representative cross-sectional surveys carried out in Brazil (2008–9), Chile (2010), Colombia (2005), Mexico (2012), Australia (2011–12), the UK (2008–16), Canada (2015) and the US (2015–16). Population attributable fractions estimated the impact of reducing ultra-processed food consumption on excessive saturated fatty acid intakes (above 10 % of total energy intake) in each country. Significant relative reductions in the percentage of excessive saturated fatty acid intakes would be observed in all countries if ultra-processed food consumption was reduced to levels observed in the first quintile's consumption. The reductions in excessive intakes ranged from 10⋅0 % (95 % CI 6⋅2–13⋅6 %) in Canada to 35⋅0 % (95 % CI 28⋅7–48⋅0 %) in Mexico. In all eight studied countries, all presenting more than 30 % of intakes with excessive saturated fatty acids, lowering the dietary contribution of ultra-processed foods to attainable, context-specific levels was shown to be a potentially effective way to reduce the percentage of intakes with excessive saturated fatty acids, which may play an important role in the prevention of non-communicable diseases, particularly cardiovascular diseases.

## Introduction

Ultra-processed foods (UPFs), according to the NOVA classification, are industrial formulations of processed food substances (oils, fats, sugars, starch, protein isolates) that contain little or no whole food and typically include flavourings, colourings, emulsifiers and other cosmetic additives^(1)^. Processes and ingredients used for the manufacture of UPFs are designed to create low-cost, highly profitable products, which are extremely palatable, convenient to use (ready-to-eat and long-lasting) and are liable to displace all other NOVA food groups (unprocessed, minimally processed and processed) and the culinary preparations made with these foods^(1)^.

Large cohort studies carried out in different countries have shown that increased dietary share of UPFs results in a higher risk of overweight/obesity, hypertension, coronary and cerebrovascular diseases, diabetes, overall and breast cancer, and all-cause mortality^(2–4)^. The positive association of diets high in UPFs with excessive energy intake and weight gain was confirmed in a randomized clinical trial^(5)^.

Although multiple mechanisms for the association between UPF intake and non-communicable diseases (NCDs) have been envisaged^(1)^ including the lower satiety potential and higher glycemic index of these foods^(1)^ and the presence of additives such as artificial sweeteners and emulsifiers^(2)^, and of compounds that are neoformed during processing or released from packaging^(2)^, the most obvious mechanism is their effect on the nutrient profile of the overall diet.

Indeed, nationally representative studies conducted in Brazil^(6)^, Chile^(7)^, Colombia^(8)^, Mexico^(9)^, Australia^(10)^, the UK^(11)^, Canada^(12)^ and the US^(13)^ have shown that increased UPF intake was associated with non-recommended intakes of most nutrients critical to NCDs, including saturated fatty acids^(14)^.

Although recent evidence suggests that the health effects of saturated fatty acids may vary depending on the type of fatty acid and on the specific food source^(15)^, a recent dietary recommendation from the World Health Organization confirmed the need of limiting saturated fatty acid intake to less than 10 % of total energy intake^(16)^. This recommendation was based on systematic review and meta-analyses of results from all randomized controlled trials and cohort studies that have examined the association between saturated fatty acid intake and incidence of and mortality by cardiovascular disease (CVD)^(16)^. Also based on systematic review and meta-analyses, the American Heart Association is still more restrictive and recommends limiting saturated fatty acid intake to 5–6 % of total energy intake^(17)^. The most recent national dietary guidelines from each of eight countries included in the present study also recommend limiting saturated fatty acid intake to reduce CVDs^(18–25)^.

The present paper describes for the first time, the proportion of excessive saturated fatty acid intake (>10 % of total energy intake) that could be potentially avoided, if UPF consumption was reduced to levels observed among the lowest consumers (the first quintile) across eight countries.

## Material and methods

### Data sources

All data used by the present study come from most recently available national dietary surveys carried out in Brazil (2008–9)^(6)^, Chile (2010)^(7)^, Colombia (2005)^(8)^, Mexico (2012)^(9)^, Australia (2011–12)^(10)^, the UK (2008–16)^(11)^, Canada (2015)^(12)^ and the US (2015–16)^(13)^. Relevant characteristics of these surveys, including age groups studied, sample sizes, dietary assessment methods and food composition tables, are described in Table 1. Briefly, UK used 4-d food diaries; Mexico and Canada used 1-d 24-h recalls, while the remaining countries used 2-d 24-h recalls. The analytical samples were restricted to participants with 3 or 4 d of food diary in the UK, one dietary recall in Mexico and Canada, two dietary recalls in Brazil and up to two dietary recalls in the remaining countries.

**Table 1.** Characteristics of nationally representative dietary surveys in eight countries

Country	Survey, year	Sample size	Age, years	Dietary assessment method	Food composition table	Sociodemographic covariates	Source
Brazil	POF, 2008–2009	34 003	10+ y	24 h recall; 2 d [means of the two studied days; only individuals who complete both days of the food record were included]	Brazilian and United States Department of Agriculture Food Composition Tables, in addition to regional recipes and food label references	Sex (male, female), age (10–19, 20–30, 31–40, 41–50, 51–59 and ≥60 years old year), schooling (up to 8 years, 9–12, over 12 years of study), race (White, Black and Other), household income per capita (fifths of income per capita), region (North, Northeast, Central-West, Southeast, South) and area (rural, urban)	Louzada *et al.*^(6)^
Chile	ENCA, 2010	4920	2+ y	24 h recall; 2 d [mean day 1 and day 2 when both days were available (20 %); day 1 otherwise]	USDA and Chilean Food Composition Tables, in addition to nutrient labels from Chilean packaged foods	Sex (male/female), age (2–19, 20–49, 50–64, ≥65 year), schooling (years of schooling of the head of the family: ≤8, 9–11, ≥12 years), income (1, 2, 3–5, ≥6 minimum wages), region (North, Centre, South, South (Austral) and Metropolitan), area (rural/urban)	Cediel *et al.*^(7)^
Colombia	ENSIN, 2005	38 643	2+ y	24 h recall; 2 d [mean day 1 and day 2 when both days were available (10 %); day 1 otherwise]	Food composition table from software developed by the School of Nutrition and Dietetics at the University of Antioquía, in Medellín, Colombia	Sex (male/female), age (2–9, 10–19, 20–34, 35–49, ≥ 50 year), income (Level 1, 2, 3 and 4), region (Atlántica, Oriental, Central, Pacífica, Bogotá, Orinoquia and Amazonia), area (rural/urban/central)	Parra *et al.*^(8)^
Mexico	ENSANUT, 2012	10 086	1+ y	24 h recall; 1 d	Food composition database compiled by the Center for Nutrition and Health Research of the INSP (Food Composition Table. Version 2014. Cuernavaca, Mexico: National Institute of Public Health; 2012)	Sex (male, female), age (1–4, 5–11, 12–19 and ≥ 20 years old), residence area (rural and urban), region (South, Central and North), socioeconomic status (low, medium and high), head of household educational level (without education, elementary education, middle school education, high school education and college graduate education)	Marrón Ponce *et al.*^(9)^
Australia	NNPAS, 2011–2012	12 153	2+ y	24 h recall; 2 d [mean day 1 and day 2 when both days were available (64 %); day 1 otherwise]	Australian AUSNUT 2011–2013 Food Composition Table specifically developed to reflect the food supply and food preparation practices during the period of the NNPAS 2011–2012	Sex (male/female), age (years, continuous), schooling (completed 9 years or below including never attended, completed 10–12 years with no graduate degree, completed 12 years with graduate degree), income (Socioeconomic Index of Disadvantage for Areas – quintiles), area (major cities of Australia, inner regional and other, which includes outer regional, remote and very remote Australia)	Machado *et al.*^(10)^
UK	NDNS, 2008–2016	12 086	1⋅5+ y	Food diary; 4 d [means of the 3/4 d. More than 91 % completed the four food diary days]	UK Department of Health's Nutrient Food Composition Table, updated for each survey year	Sex (male, female), age (years, continuous), ethnicity (White, Mixed ethnic group, Black or Black British, Asian or Asian British and Other race), region (England North, England Central/Midlands, England South (including London), Scotland, Wales and Northern Ireland), survey year (years 1–6) and equivalised household income (equivalised for different household sizes and composition using the McClements equivalence scale)	Rauber *et al.*^(11)^
Canada	CCHS, 2015	20 478	1+ y	1 d; 24 h recall	Canadian Nutrient File (CNF), 2015	Sex (male/female), age (continuous in years), schooling (there are four categories in this variable: (1) below high school diploma, (2) high school diploma or equivalent, (3) trade certificate or college degree, CEGEP degree, university certificate, university degree below BA degree, and (4) university degree and above), birthplace (Canada or not), zone (urban or rural)	Moubarac *et al.*^(12)^
US	NHANES, 2015–2016	8113	1+ y	24 h recall; 2 d [mean day 1 and day 2 when both days were available (82⋅4 %); day 1 otherwise]	United States Department of Agriculture Food Composition Table, updated for each survey year	Sex (male/female), age (1–5, 6–11, 12–19, 20–39, 40–59, 60+ years), race/ethnicity (Mexican-American, Other Hispanic, Non-Hispanic White, Non-Hispanic Black, Other Race including Multi-Racial) and ratio of family income to poverty (categorised based on Supplemental Nutrition Assistance Program (SNAP) eligibility as 0⋅00–1⋅30, >1⋅30–3⋅50 and >3⋅50 and above)	Martinez *et al.*^(13)^

The present study was conducted according to the guidelines laid down in the Declaration of Helsinki, and all procedures involving human subjects were approved by the Research Ethics Committee from the Faculdade de Saúde Pública at the Universidade de São Paulo (Protocol 128958, 19 October 2012); Comité de Ética de Investigación de la Escuela de Medicina de la Pontificia Universidad Católica de Chile; Comité de ética de la Universidad del Rosario (Bogotá, Colombia); INSP Research, Biosafety, and Ethics Committee in Cuernavaca, Mexico; Australian Government Department of Health and Ageing Departmental Ethnics Committee in 2011; UK relevant research ethics and governance committees; Comité d’éthique de la recherche en sciences et en santé (CERSES) of Université de Montréal (17-017-CERES-D); US NCHS Research Ethics Review Board (ERB) Approval (Continuation of Protocol #2011–17). Written informed consent was obtained from all subjects.

### Data analysis

All analyses were run separately for each country using mean of 3 or 4 d of food diaries in the UK, day 1 dietary recall data in Mexico and Canada, mean day 1 and day 2 dietary recall data in Brazil, and mean day 1 and day 2 dietary recall data if available and day 1 otherwise in the remaining countries. Continuous variables presented an approximately normal distribution. First, we calculated the mean percentage of energy intake from UPFs (classified using the NOVA system)^(1)^, the mean percentage of energy intake from saturated fatty acids and the percentage of intakes with excessive saturated fatty acid (>10 % of energy intake, according to WHO recommendation)^(14,16)^. Then, we used linear regression models to estimate the association between the dietary energy contribution of UPFs (categorised into quintiles) and the dietary content of saturated fatty acids (expressed as percentage of total energy). Poisson regression models with robust variance were used to assess whether the percentage of intakes with excessive saturated fatty acid increased across quintiles of the dietary energy contribution of UPFs.

To evaluate dose-response associations, tests of linear trend were performed by treating quintiles of UPF consumption as an ordinal (1–5) variable.

Finally, we estimated, again for each country, the UPF population attributable fraction (PAF) defined as the proportion of intakes with excessive saturated fatty acid intake that would be potentially avoided if UPF consumption (the exposure) was reduced to attainable values. We assumed attainable values to be those estimated in the first quintile of UPF consumption for each country.

We calculated PAF^(26,27)^ through the following equation:
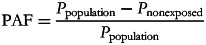
where *P*_population_ is the percentage of intakes with excessive saturated fatty acid in the total population and *P*_nonexposed_ is the percentage of intakes with excessive saturated fatty acid in the first quintile of UPF consumption. All models were adjusted for the sociodemographic covariates displayed in Table 1. Statistical hypotheses were tested using a two-tailed *P* ≤ 0⋅05 level of significance. All analyses were conducted with Stata statistical software package version 14.

## Results

Table 2 describes overall energy contributions of UPF and saturated fatty acid intakes across the eight studied countries. The mean energy contribution of UPFs varied from 15⋅9 % (95 % CI 15⋅3, 16⋅5) in Colombia to 56⋅7 % (95 % CI 56⋅2, 57⋅1) in the UK. Colombia showed the lowest mean dietary intake of saturated fatty acids (8⋅6 % (95 % CI 8⋅4, 8⋅8) of total energy intake) and the lowest percentage of intakes with excessive saturated fatty acids (31⋅40 % (95 % CI 31⋅38, 31⋅42)) while the highest estimates for these two parameters were observed in the UK (12⋅1 % (95 % CI 12⋅0, 12⋅2) and 74⋅0 % (95 % CI 72⋅8, 75⋅2), respectively).

**Table 2.** Contribution of UPFs and saturated fatty acids to total energy intake (kcal/d) and prevalence of excessive saturated fatty acid intakes in eight countries

Country	Total energy intake (kcal/d)	Percentage of total energy intake from UPFs	Percentage of total energy intake from saturated fatty acids	Prevalence of intakes with excessive saturated fatty acids
	mean (95 % CI)	mean (95 % CI)	mean (95 % CI)	% (95 % CI)
Colombia	1835⋅1 (1811⋅6, 1858⋅6)	15⋅9 (15⋅3, 16⋅5)	8⋅6 (8⋅4, 8⋅8)	31⋅40 (31⋅38, 31⋅42)
Brazil	1952⋅9 (1932⋅5, 1973⋅3)	18⋅9 (18⋅2, 19⋅2)	9⋅7 (9⋅6, 9⋅8)	40⋅8 (39⋅7, 41⋅9)
Chile	1804⋅7 (1760⋅6, 1848⋅8)	28⋅6 (27⋅7, 29⋅6)	8⋅8 (8⋅7, 9⋅0)	32⋅4 (30⋅2, 34⋅7)
Mexico	1923⋅4 (1886⋅2, 1960⋅6)	30⋅0 (29⋅2, 30⋅8)	11⋅2 (11⋅0, 11⋅4)	58⋅4 (56⋅5, 60⋅3)
Australia	1926⋅5 (1908⋅5, 1944⋅5)	41⋅6 (41⋅1, 42⋅1)	12⋅0 (11⋅8, 12⋅1)	67⋅1 (66⋅0, 68⋅2)
Canada	1816⋅1 (1804⋅7, 1827⋅5)	51⋅0 (50⋅6, 51⋅4)	11⋅0 (10⋅9, 11⋅1)	56⋅80 (56⋅79, 56⋅81)
US	1995⋅1 (1956⋅9, 2033⋅4)	56⋅2 (55⋅0, 57⋅3)	11⋅5 (11⋅3, 11⋅7)	66⋅9 (64⋅2, 69⋅6)
UK	1746⋅7 (1732⋅9 1760⋅6)	56⋅7 (56⋅2, 57⋅1)	12⋅1 (12⋅0, 12⋅2)	74⋅0 (72⋅8, 75⋅2)

**Fig. 1.**
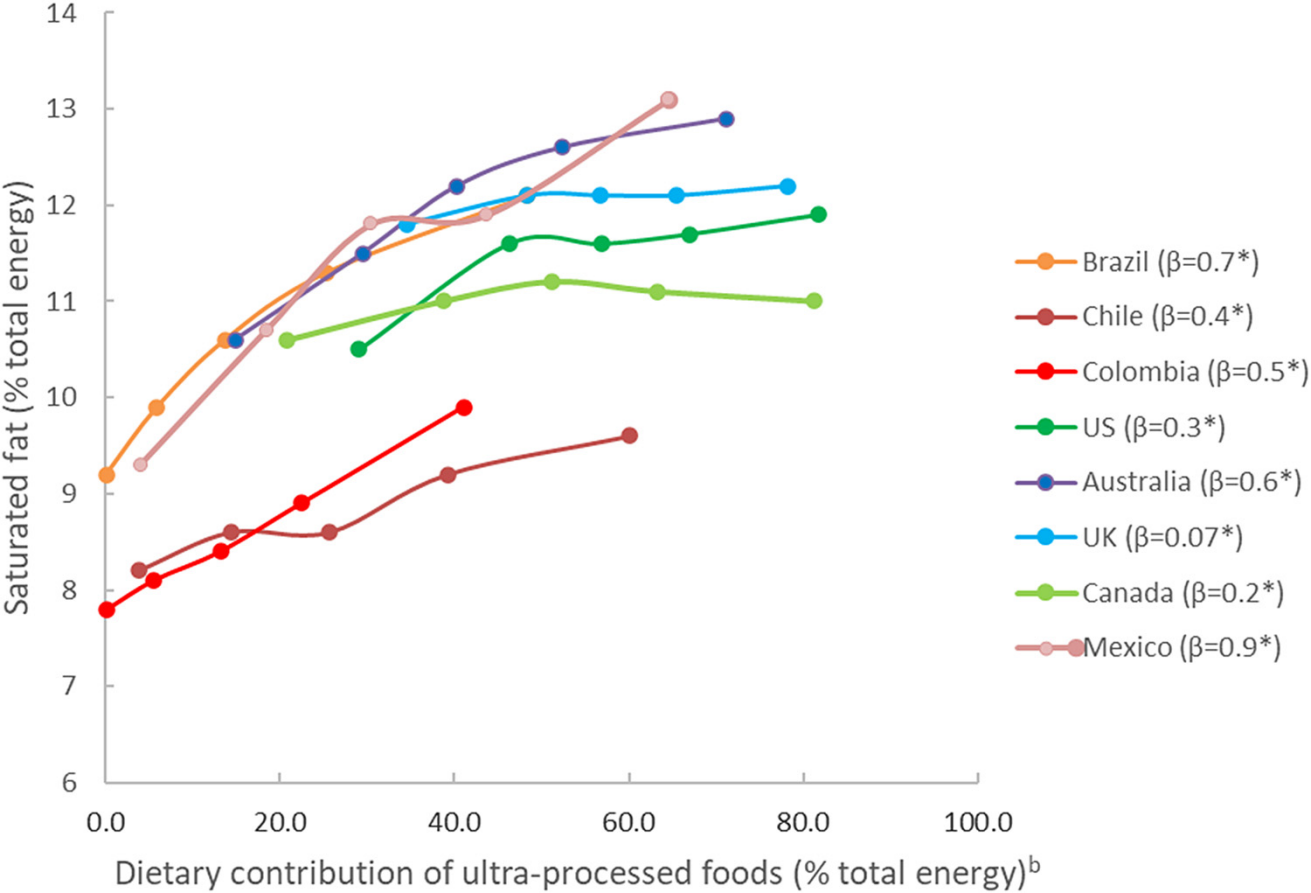
Adjusted^a^ mean dietary content of saturated fatty acids across quintiles of the dietary contribution of UPFs in eight countries. ^a^Adjusted according to covariates listed in Table 1. ^b^Knots corresponding to country-specific quintiles of the dietary contribution of UPFs. *Statistically significant coefficient for linear trend (*P* < 0⋅05).

As depicted in Fig. 1, the adjusted mean dietary intake of saturated fatty acids increased significantly across quintiles of the dietary contribution of UPFs in all countries (*P* for linear trend <0⋅05). Supplementary Table S1 of Supplementary material describes the mean and range of the dietary contribution of UPFs per quintile.

**Fig. 2.**
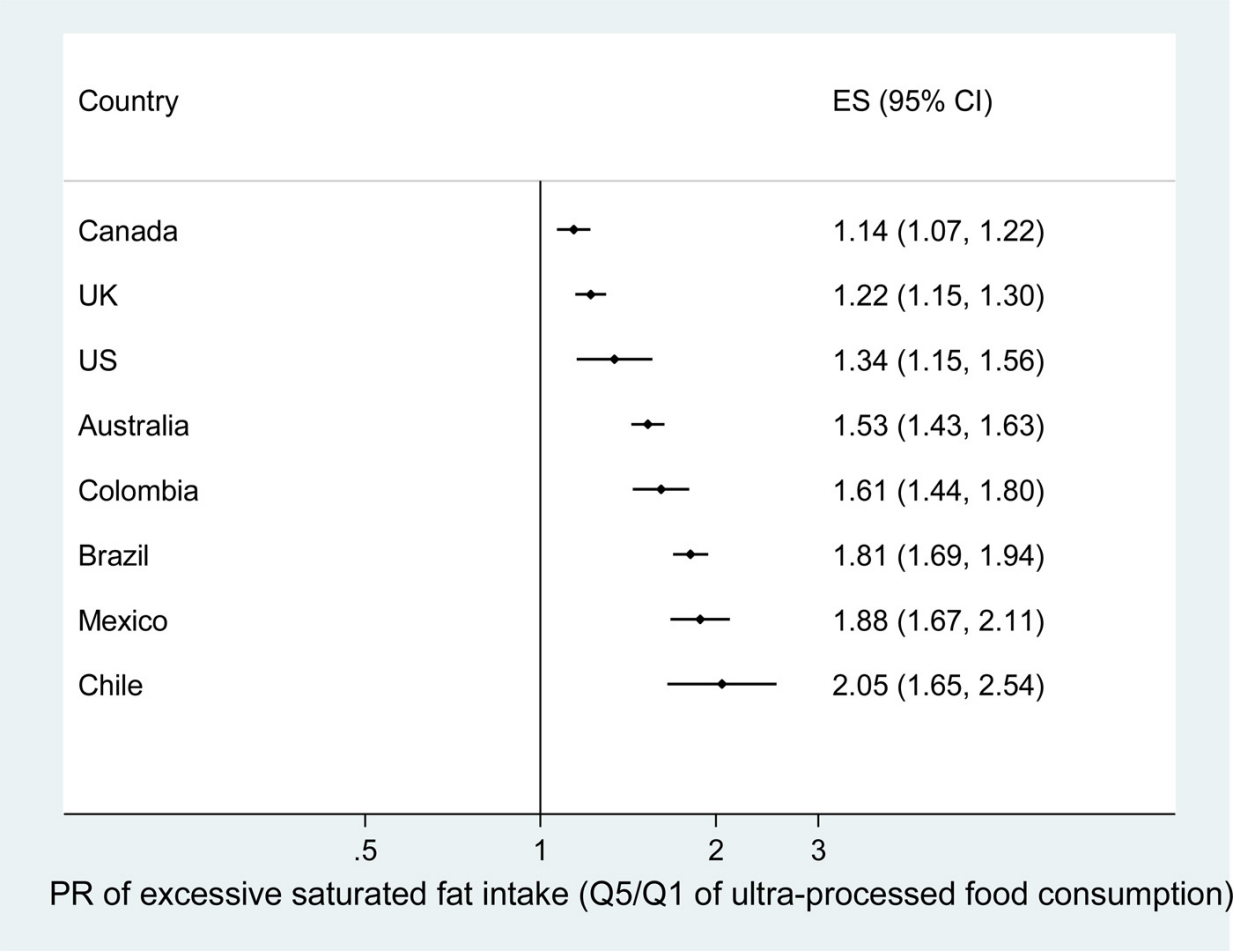
Adjusted^a^ PR of excessive saturated fatty acid intake (>10 %) between extreme quintiles of the dietary contribution of UPFs in eight countries. ^a^Adjusted according to covariates listed in Table 1.

Table 3 shows the adjusted percentage of excessive saturated fatty acid intakes according to quintiles of the dietary contribution of UPFs. In all eight countries, the percentage of intakes with excessive saturated fatty acids increased significantly across the quintiles (*P* for linear trend <0⋅001). The adjusted ratio of the percentage of intakes with excessive saturated fatty acid between extreme UPF quintiles ranged from 1⋅14 (95 % CI 1⋅07, 1⋅22) in Canada up to 2⋅05 (95 % CI 1⋅65, 2⋅54) in Chile (Fig. 2). The values in the first *v*. the last quintile almost doubled in Brazil, Mexico and Chile.

**Table 3.** Adjusted^a^ percentage of intakes with excessive saturated fatty acids (>10 %) across quintiles of the dietary contribution of UPFs in eight countries

Country	Quintiles of the dietary contribution of UPFs^a^					
	Q1	Q2	Q3	Q4	Q5	
Colombia	24⋅9 (22⋅9; 26⋅9)	27⋅1 (25⋅1; 29⋅1)	29⋅4 (27⋅4; 31⋅4)	33⋅8 (31⋅8; 35⋅8)	40⋅2 (38⋅2; 42⋅2)	*
Brazil	31⋅6 (29⋅6; 33⋅6)	32⋅6 (30⋅7; 34⋅5)	36⋅1 (34⋅1; 38⋅1)	44⋅3 (42⋅1; 46⋅5)	57⋅4 (55⋅0; 59⋅7)	*
Chile	22⋅5 (18⋅7; 26⋅3)	26⋅8 (21⋅7; 31⋅9)	27⋅6 (23⋅2; 32⋅0)	37⋅9 (33⋅0; 42⋅8)	46⋅2 (40⋅3; 52⋅0)	*
Mexico	38⋅6 (34⋅5; 42⋅7)	51⋅8 (48⋅2; 55⋅4)	64⋅2 (60⋅6; 67⋅7)	67⋅7 (63⋅9; 71⋅6)	72⋅5 (69⋅0; 75⋅9)	*
Canada	51⋅40 (51⋅38; 51⋅42)	56⋅60 (56⋅58; 56⋅62)	58⋅90 (58⋅88; 58⋅92)	58⋅20 (58⋅18; 58⋅22)	58⋅60 (58⋅58; 58⋅62)	*
Australia	50⋅4 (47⋅6; 53⋅1)	62⋅9 (60⋅2; 65⋅6)	69⋅2 (66⋅7; 71⋅6)	75⋅1 (72⋅9; 77⋅4)	77⋅3 (75⋅1; 79⋅6)	*
US	53⋅8 (47⋅5; 60⋅1)	67⋅1 (63⋅0; 71⋅1)	69⋅5 (66⋅6; 72⋅5)	71⋅9 (69⋅1; 74⋅6)	71⋅9 (67⋅6; 76⋅2)	*
UK	65⋅5 (62⋅3; 68⋅7)	73⋅3 (70⋅5; 76⋅1)	76⋅1 (73⋅5; 78⋅8)	75⋅7 (73⋅1; 78⋅3)	80⋅1 (77⋅7; 82⋅4)	*

a Adjusted according to covariates listed in Table 1. 95 % CI in parenthesis.

* Statistically significant linear trend (*P*≤0⋅05).

**Fig. 3.**
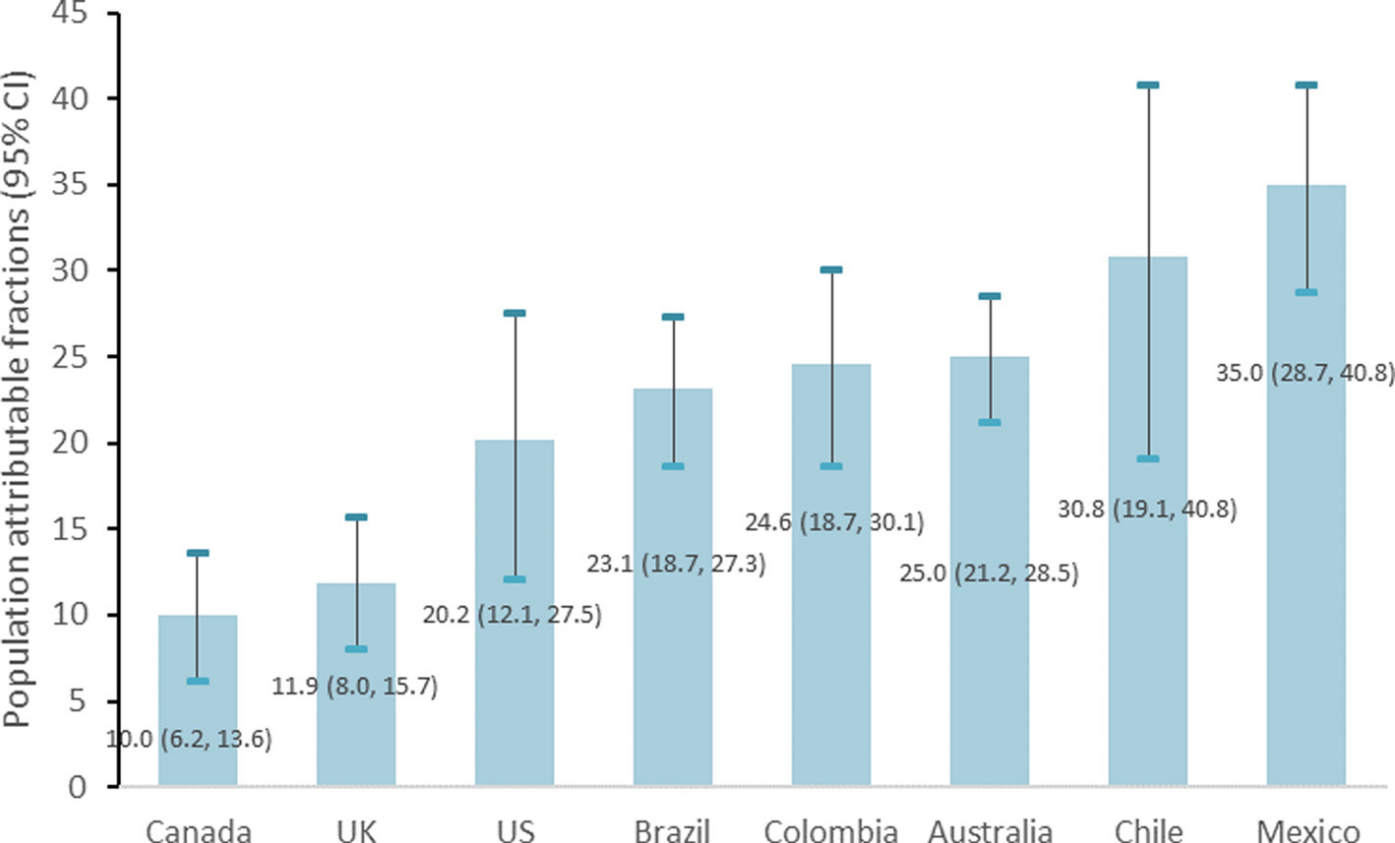
Impact of UPF consumption on the adjusted^a^ prevalence of excessive saturated fatty acid intake in eight countries. ^a^Adjusted according to covariates listed in Table 1.

If the dietary contribution of UPFs was reduced to levels observed in the first quintile of each country, statistically significant reductions in the percentage of excessive saturated fatty acid intakes would be observed in all eight countries. The reductions would vary from 10⋅0 % (95 % CI 6⋅2, 13⋅6) in Canada all the way up to 35⋅0 % (95 % CI 28⋅7, 40⋅8) in Mexico (Fig. 3).

## Discussion

In the present study across eight middle- and high-income countries, we observed that the rise in UPF consumption was systematically associated with an increase in both the mean saturated fatty acid intakes and in the percentage of excessive intakes. The percentage of excessive saturated fatty acid intakes would be significantly reduced in all countries (between 10 % in Canada and 35 % in Mexico), if UPF consumption was decreased to context-specific attainable levels such as those estimated for the lowest quintile of UPF consumption in each country.

Despite the fact that not all UPF items are a source of saturated fatty acids and some of them are even exempt of fat, soft drinks being an example, our findings show that, irrespective of the country, dietary patterns characterised by relatively higher overall contribution of UPFs tend to have a higher content of saturated fatty acids. One likely explanation is the frequent use of saturated fatty acid rich crude palm oil (51 % content of saturated fatty acids) and palm kernel oil (84 % content of saturated fatty acids)^(28)^ in the manufacture of several UPFs such as baked goods, candies, cakes, cheese analogues, chips, chocolate, confectionary fats, cookies, crackers, doughnuts, frozen meals (pancakes, pies, pizza, potatoes), ice cream, instant noodles, margarines, shortenings and other butter substitutes, microwave popcorn, non-dairy creamers, salad dressings, snacks, soups, among others^(29,30)^. The preference for these oils and their fractions and blends lies in their fragrance and neutral taste and in their capacity to confer the texture obtained with the use of butter or lard (without the need of using hydrogenation, thus reducing trans-fat content) as well as to increase the thermal and oxidative stability of the fat, and thus the shelf life of the final product^(28,29)^. The high productivity of palm oil, which is able to produce up to ten times more oil per hectare of plantation than other leading oilseed crops^(31)^, may be an additional economic force driving its use in UPFs. In fact, in 2012, crude palm oil and palm kernel oil (both extracted from palm fruit) overtook soybean oil as the most important vegetable oil in the world, accounting for 32 % of global fats and oils production, 90 % of which being used by the edible food industry^(29,31)^.

The relationship found between UPF consumption and excessive saturated fatty acid intake indicates that these intakes may be one of the mechanisms for the prospective association between UPF consumption and both cardiovascular diseases^(2,32)^ and several major risk factors for these diseases, including overweight and obesity, hypertension, dyslipidaemia and diabetes^(2,3,33)^.

Taxes, front-of-pack labels and dietary guidelines are potential vehicles that can be used to discourage the consumption of UPFs. However, while reducing UPF consumption to levels observed in the first quintile would help, this is not enough to eliminate excessive saturated fatty acid intake. Australia, UK, Canada and US would still have over 50 % percentage of excessive intakes, Brazil and Mexico over 30 %, and Chile and Colombia over 20 %. Especially in the UK and US but also in Australia and Canada, with already significant UPF consumption in the first quintile, countries would need to set goals to reduce UPFs well below levels observed in the first quintile. In Brazil, Mexico, Chile and Colombia, in which mean UPF contribution is already below 5 % in the first quintile, countries would need to extend the reduction to non-ultra-processed sources of saturated fatty acids such as meat, butter and cheese.

On the other hand, in all eight countries evaluated in the present study, UPF consumption was shown to be inversely related to protein and fibre intakes and directly related to free sugar intake^(6–13)^ suggesting that UPFs tend to replace healthier sources of food and that reducing its consumption would probably entail additional cardiovascular and other health benefits.

Strengths of the present study include the use of large, nationally representative samples of the population of eight middle- and high-income countries, maximising external validity of results. The use of individual consumption data rather than market disappearance or household purchasing data, which may under- or overestimate amounts consumed, is an additional strength of the present study as well as the fact that the NOVA classification was applied similarly across all countries after disaggregating handmade recipes into underlying ingredients, maximising the comparability across settings. The use of context-specific scenarios across the countries for estimating the reduction in the percentage of intakes with excessive fatty acids is also a strength of the present study.

Though the most recent surveys were used for each country, the years of data collection were less recent in some countries possibly not reflecting actual consumption and also limiting comparisons across countries. Despite existing evidence of the increasing trends in UPF consumption in many countries^(34)^, how this should affect the population attributable fractions is less clear because it depends much more on the difference in risk between the first and all other quintiles than on the absolute contribution of UPFs to the diet. Though sample sizes varied from country to country ranging from 4920 in Chile to 38 643 in Colombia, all provided statistically significant and relatively precise PAFs (see Fig. 3). Random and systematic error may bias self-reported dietary intake data collected through 24-h recall/food diaries, especially regarding absolute energy intakes, even though the applied standardised methods should contribute to minimising it, and also the fact that we always used relative estimates. Though social desirability bias may lead to an underestimation of the dietary contribution of UPFs and of saturated fatty acids, this would most likely not affect the association between the two variables or it would bias the association towards the null. Despite these limitations inherent to self-reported diet, 24-h recalls/food diaries are considered the least biased method and the best for describing dietary intake at the population level^(35)^. In most countries, the assessment of dietary intakes was exclusively or mostly based on 1 d recalls potentially overestimating the frequency of individuals with very low or very high long-term saturated fatty acid intakes^(36)^. Though most countries included participants aged 1 or 2 years onwards, in Brazil only participants aged 10 years and older were included. If as shown in other countries such as the US^(37)^, children consume more UPFs than adults, UPF consumption, saturated fatty acids and thus, PAF, of the overall population may be underestimated in Brazil.

In addition, although all studies collected some information indicative of the type of processing to which foods were submitted before consumption or culinary preparation (i.e. place of meals and product brands), this information was not available for all food items, which could lead to modest over- or underestimation of UPF consumption. An additional source of error may be the use of combined foreign and national food composition tables in some countries (i.e. Brazil and Chile). Also, reformulation could have decreased saturated fatty acid content of UPFs since data collection, though the fact that associations persisted in studies with more recent data suggests that this is unlikely the case. Finally, PAFs need to be interpreted with caution especially because these are applied to population estimates and do not account for individual-level behaviour changes including food substitutions, replacements and abandonment effects associated with the reduction in UPF consumption.

In summary, the present study, carried out in nationally representative samples of the population of eight middle- and high-income countries, indicates that lowering the dietary contribution of UPFs to attainable, context-specific levels is a potentially effective way to reduce saturated fatty acid intake and the percentage of intakes with excessive content, which may play an important role in the prevention of NCDs, particularly cardiovascular diseases.

## Supplementary material

For supplementary material accompanying this paper visit http://dx.doi.org/10.1017/jns.2021.30. click here to view supplementary material

## References

[1] Monteiro CA, Cannon G, Levy RB, (2019) Ultra-processed foods: What they are and how to identify them. Public Health Nutr 22, 936–941. doi:10.1017/S1368980018003762. 30744710 PMC10260459

[2] Srour B, Fezeu LK, Kesse-Guyot E, (2019) Ultra-processed food intake and risk of cardiovascular disease: Prospective cohort study (NutriNet-Santé). Br Med J 365, l1451. doi:10.1136/bmj.l1451. 31142457 PMC6538975

[3] Srour B, Fezeu LK, Kesse-Guyot E, (2019) Ultraprocessed food consumption and risk of type 2 diabetes among participants of the NutriNet-Santé prospective cohort. JAMA Intern Med. doi:10.1001/jamainternmed.2019.5942. PMC6990737 31841598

[4] Schnabel L, Kesse-Guyot E, Allès B, (2019) Association between ultraprocessed food consumption and risk of mortality among middle-aged adults in France. JAMA Intern Med 179, 490–498. doi:10.1001/jamainternmed.2018.7289. 30742202 PMC6450295

[5] Hall KD, Ayuketah A, Brychta R, (2019) Ultra-processed diets cause excess calorie intake and weight gain: An inpatient randomized controlled trial of *ad libitum* food intake. Cell Metab 30, 67–77.e3. doi:10.1016/j.cmet.2019.05.008. Epublication 6 May 2019. 31105044 PMC7946062

[6] Costa Louzada ML, Martins AP, Canella DS, (2015) Ultra-processed foods and the nutritional dietary profile in Brazil. Rev Saude Publica 49, 38. doi:10.1590/S0034-8910.2015049006132. 26176747 PMC4544452

[7] Cediel G, Reyes M, Corvalán C, (2020) Ultra-processed foods drive to unhealthy diets: Evidence from Chile. Public Health Nutr, 1–10. [Epub ahead of print]. doi:10.1017/S1368980019004737. PMID: 32338229. PMC10195482 32338229

[8] Parra DC, da Costa-Louzada ML, Moubarac JC, (2019) Association between ultra-processed food consumption and the nutrient profile of the Colombian diet in 2005. Salud Publica Mex 61, 147–154. doi:10.21149/9038. 30958957

[9] Marrón-Ponce JA, Flores M, Cediel G, (2019) Associations between consumption of ultra-processed foods and intake of nutrients related to chronic non-communicable diseases in Mexico. J Acad Nutr Diet 119, 1852–1865. doi:10.1016/j.jand.2019.04.020. 31262695

[10] Machado PP, Martinez Steele E, Levy R, (2019) Ultra-processed foods and recommended intake levels of nutrients linked to non-communicable diseases in Australia: Evidence from a nationally representative cross-sectional study. BMJ Open 9, e029544. doi:10.1136/bmjopen-2019-029544. PMC6720475 31462476

[11] Rauber F, da Costa Louzada ML, Steele EM, (2018) Ultra-processed food consumption and chronic non-communicable diseases-related dietary nutrient profile in the UK (2008–2014). Nutrients 10. doi:10.3390/nu10050587. PMC5986467 29747447

[12] Moubarac JC, Batal M, Louzada ML, (2017) Consumption of ultra-processed foods predicts diet quality in Canada. Appetite 108, 512–520. doi:10.1016/j.appet.2016.11.006. Epublication 4 November 2016. 27825941

[13] Martínez Steele E, Popkin BM, Swinburn B, (2017) The share of ultra-processed foods and the overall nutritional quality of diets in the US: Evidence from a nationally representative cross-sectional study. Popul Health Metr 15, 6. doi:10.1186/s12963-017-0119-3. 28193285 PMC5307821

[14] World Health Organization (2018) Healthy Diet. Fact Sheet No. 394. Geneva: World Health Organization.

[15] Astrup A, Bertram HC, Bonjour JP, (2019) WHO draft guidelines on dietary saturated and trans fatty acids: Time for a new approach? BMJ 366, l4137. doi:10.1136/bmj.l4137. 31270106

[16] WHO (2018) https://extranet.who.int/dataform/upload/surveys/666752/files/Draft%20WHO%20SFA-TFA%20guidelines_04052018%20Public%20Consultation(1).pdf, 27/May/2021.

[17] Van Horn L, Carson JAS, Appel LJ, (2016) Recommended dietary pattern to achieve adherence to the American Heart Association/American College of Cardiology (AHA/ACC) guidelines: A scientific statement from the American Heart Association. Circulation 134, e505–e529. doi:10.1161/CIR.0000000000000462. 27789558

[18] National Health and Medical Research Council (2013) Australian Dietary Guidelines. Canberra, Australia: National Health and Medical Research Council.

[19] Guia Alimentar para a População Brasileira [Dietary Guidelines for the Brazilian population]. Brasília – DF (2014).

[20] Public Health England (2019) Canada's Dietary Guidelines for Health Professionals and Policy Makers. Her Majesty the Queen in Right of Canada, as represented by the Minister of Health.

[21] Dietary guidelines for the Chilean population. Ministry of Health, May 16th 2013.

[22] Guías Alimentarias para la población Colombiana mayor de 2 años Basadas en Alimentos [Food-based dietary guidelines for the Colombian population over 2 years of age]. Noviembre (2015).

[23] Guías alimentarias y de actividad física en contexto de sobrepeso y obesidad en la población mexicana [Dietary and physical activity guidelines in the context of overweight and obesity in the Mexican population]. Marzo de (2015).

[24] https://www.gov.uk/government/publications/the-eatwell-guide.

[25] Dietary Guidelines for Americans 2015–2020, 8th ed.

[26] Steenland K & Armstrong B (2006) An overview of methods for calculating the burden of disease due to specific risk factors. Epidemiology 17, 512–519. 16804473 10.1097/01.ede.0000229155.05644.43

[27] Rezende LFM & Eluf-Neto J (2016) Population attributable fraction: Planning of diseases prevention actions in Brazil. Rev Saúde Pública 50, 30. 10.1590/S1518-8787.2016050006269 PMC4902656 27305404

[28] Vaclavik VA & Christian EW (2014) Essentials of Food Science, 4th ed., US: Springer.

[29] Mba OI, Dumont MJ & Ngadi M (2015) Palm oil: Processing, characterization and utilization in the food industry – A review. Food Bioscience 10, 26–41. doi:10.1016/j.fbio.2015.01.003.

[30] Mancini A, Imperlini E, Nigro E, (2015) Biological and nutritional properties of palm oil and palmitic acid: Effects on health. Molecules 20, 17339–17361, published online 18 September 2015. doi:10.3390/molecules200917339. 26393565 PMC6331788

[31] Oil World (2013) Oil world annual 2013. http://www.oilworld.biz/app.php. (released June 2013).

[32] Bonaccio M, Di Castelnuovo A, Costanzo S, (2020) Consumption of ultra-processed foods and beverages is associated with increased risk of cardiovascular mortality in the Moli-sani Study Cohort. Circulation 141, A49. 10.1093/ajcn/nqaa299 33333551

[33] Rauber F, Campagnolo PD, Hoffman DJ, (2015) Consumption of ultra-processed food products and its effects on children's lipid profiles: A longitudinal study. Nutr Metab Cardiovasc Dis 25, 116–122. doi:10.1016/j.numecd.2014.08.001. 25240690

[34] Vandevijvere S, Jaacks LM, Monteiro CA, (2019) Global trends in ultraprocessed food and drink product sales and their association with adult body mass index trajectories. Obes Rev 20, 10–19. doi:10.1111/obr.12860. Epublication 17 May 2019. 31099480

[35] Prentice RL, Mossavar-Rahmani Y, Huang Y, (2011) Evaluation and comparison of food records, recalls, and frequencies for energy and protein assessment by using recovery biomarkers. Am. J. Epidemiol 174, 591–603. 21765003 10.1093/aje/kwr140 PMC3202154

[36] Herrick KA, Rossen LM, Parsons R, (2018) Estimating usual dietary intake from national health and nutrition examination survey data using the National Cancer Institute method. Vital Health Stat 2 (178), National Center for Health Statistics. 29775432

[37] Baraldi LG, Steele EM, Canella DS & Monteiro CA (2018) Consumption of ultra-processed foods and associated sociodemographic factors in the USA between 2007 and 2012: Evidence from a nationally representative cross-sectional study. BMJ Open 8, e020574. doi:10.1136/bmjopen-2017-020574. PMC5855172 29525772

