# Supplementary material for: The burden of excessive saturated fatty acid intake attributed to ultra-processed food consumption: a study conducted with nationally representative cross-sectional studies from eight countries
Source: J Nutr Sci. 2021 Jun 4;10:e43. doi: 10.1017/jns.2021.30 (PMC8190718; doi:10.1017/jns.2021.30)
Supplement: Supplementary file 1 [file S2048679021000306sup001.docx]

Supplementary Table 1. Mean dietary contribution of ultra-processed foods across quintiles of the dietary contribution of ultra-processed foods in eight countries.

| **Country** | **Mean (min max values) of dietary contribution of ultra-processed foods (% of total energy)** | | | | |
| --- | --- | --- | --- | --- | --- |
|  | **Q1** | **Q2** | **Q3** | **Q4** | **Q5** |
| **Brazil** | 0.1 (0 to 4.9) | 5.9 (4.9 to 12.4) | 13.7 (12.4 to 21.3) | 25.3 (21.3 to 34.2) | 48.4 (34.2 to 98.3) |
| **Chile** | 3.8 (0 to 9.3) | 14.4 (9.3 to 19.9) | 25.7 (19.9 to 31.7) | 39.2 (31.7 to 47.4) | 60.1 (47.5 to 100) |
| **Colombia** | 0.2 (0 to 1.6) | 5.6 (1.6 to 9.3) | 13.2 (9.3 to 17.2) | 22.5 (17.2 to 28.7) | 41.1 (28.7 to 100) |
| **Mexico** | 4.1 (0 to 11.8) | 18.5 (11.8 to 24.6) | 30.5 (24.6 to 36.8) | 43.8 (36.8 to 51.7) | 64.6 (51.7 to 100) |
| **Australia** | 14.9 (0 to 23.6) | 29.5 (23.6 to 34.8) | 40.3 (34.8 to 45.8) | 52.3 (45.8 to 59.4) | 71.2 (59.4 to 100) |
| **UK** | 34.6 (1.8 to 43.3) | 48.4 (43.3 to 52.7) | 56.8 (52.7 to 60.9) | 65.4 (60.9 to 70.1) | 78.2 (70.1 to 100) |
| **Canada** | 20.9 (0 to 31.5) | 38.8 (31.5 to 45.2) | 51.2 (45.2 to 56.7) | 63.2 (56.7 to 69.9) | 81.2 (69.9 to 100) |
| **US** | 29.1 (0 to 39.9) | 46.3 (40.0 to 52.0) | 56.9 (52.0 to 61.7) | 66.9 (61.7 to 72.5) | 81.7 (72.5 to 100) |
